# Candida species identified by MALDI-TOF and antifungal susceptibility in hospitalized patients with COVID-19 in Peru

**DOI:** 10.17843/rpmesp.2026.431.15685

**Published:** 2026-03-27

**Authors:** Rolando Paredes-Gago, Sorell Alvarado-Vela, Clelia Céspedes-Román

**Affiliations:** 1 National Institute of Health, National Center for Public Health, Lima, Peru.

**Keywords:** Candida, COVID-19, Antifungal Drug Resistance, Opportunistic Infections

## Abstract

**Objectives.:**

To identify *Candida* species using MALDI-TOF and analyze their antifungal susceptibility from *Candida* genus isolates in hospitalized patients with COVID-19 between November 2020 and April 2022.

**Materials and methods.:**

An observational, descriptive, and cross-sectional study based on the secondary analysis of microbiological and clinical-epidemiological data from 260 *Candida* isolates, primarily from urine, respiratory secretions, and blood cultures, referred to the National Reference Laboratory of Mycology of the National Institute of Health. Identification was performed using phenotypic techniques and MALDI-TOF, and antifungal susceptibility was evaluated using disk diffusion and broth microdilution according to Clinical and Laboratory Standards Institute (CLSI) criteria.

**Results.:**

*Candida albicans* was the most prevalent species (64.6%), followed by *Candida tropicalis*, *Candida glabrata*, *Candida parapsilosis*, and *Candida auris*. Most isolates showed susceptibility to voriconazole and caspofungin, while varying percentages of susceptibility to fluconazole were observed, especially in *C. glabrata* and *C. auris*. Minimum inhibitory concentration (MIC) values showed variability between species and antifungals.

**Conclusions.:**

Non-*albicans* species represented a considerable proportion of the analyzed isolates. These findings describe the pattern of species and antifungal susceptibility in strains referred to the National Reference Laboratory of Mycology of the National Institute of Health during the COVID-19 pandemic.

## INTRODUCTION

During the COVID-19 pandemic, an increase in fungal infections was observed in critical patients, especially in intensive care units (ICU) ^1^^,^^2^. Although *Candida* infections have traditionally been considered opportunistic, in severe cases, the infection appears to be more related to factors derived from clinical management than to classic states of primary immunosuppression. In particular, invasive candidiasis has been associated with prolonged use of broad-spectrum antibiotics and corticosteroids, mechanical ventilation, the presence of invasive devices, and prolonged hospital stay, conditions that favor colonization and infection by *Candida*^3^^-^^5^. Various studies have documented the presence of species such as *Candida albicans*, *Candida glabrata*, *Candida tropicalis*, *Candida parapsilosis*, and *Candida auris* in patients with COVID-19, in both colonization and invasive infection cases ^6^.

Candidemia has been reported in up to 8.9% of patients with COVID-19 in the ICU, presenting high mortality rates and the emergence of antifungal-resistant strains, including fluconazole and voriconazole ^7^^-^^9^. These variations in species distribution and susceptibility profiles suggest changes in the epidemiology of COVID-19-associated candidemia, with relevant therapeutic implications. In Peru, information regarding *Candida* species and their antifungal susceptibility profiles is limited. A multicenter study conducted in 15 hospitals analyzed 73 blood culture isolates, finding that *C. parapsilosis* (53.4%), *C. albicans* (31.5%), and *C. tropicalis* (6.8%) were the most prevalent species, and that all those isolates were susceptible to amphotericin B, fluconazole, voriconazole, and anidulafungin ^10^. In the Lambayeque region, it was reported that 20% of isolates other than *C. albicans* showed resistance to fluconazole, and 22% to voriconazole ^11^. During the pandemic, the finding of fluconazole-resistant *C. auris* was reported for the first time in Peru in three patients hospitalized in the ICU, suggesting an emerging change in local epidemiology ^12^. In Lima and Callao, an incidence of 2.04 cases per 1000 admissions was documented, with a predominance of non-*albicans* species (72%) and low resistance to fluconazole (<3%) ^13^.

Although mycological culture is widely available, access to identification methods at the species level and antifungal susceptibility testing is limited outside of Lima. Furthermore, the availability of broad-spectrum antifungals, particularly echinocandins, is restricted in several health facilities ^14^, which may hinder the early detection of emerging species and resistance profiles.

The massive and indiscriminate use of antimicrobials during the pandemic, together with hospital overcrowding conditions, is considered to have favored the selection of resistant strains and the displacement of less common species. Likewise, alterations in the respiratory and digestive microbiota induced by intensive treatments could favor the overgrowth of yeasts and their subsequent tissue invasion.

The present study seeks to provide evidence on the distribution of *Candida* species and their antifungal susceptibility profiles in a context of high hospital pressure, generating information for the surveillance of healthcare-associated fungal infections by providing local data to monitor the trend of emerging species and antifungal susceptibility patterns and guide fungal infection control and prevention strategies. In this context, the study aimed to identify species of the genus *Candida* using the MALDI-TOF method and analyze their antifungal susceptibility in isolates obtained from hospitalized patients with COVID-19 between November 2020 and April 2022.

**KEY MESSAGES**

Motivation for the study. During the COVID-19 pandemic, an increase in opportunistic fungal infections was reported; however, in Peru, extensive descriptive studies documenting *Candida* species and their antifungal susceptibility in the hospital context are not available.

Main findings. The findings demonstrated that *C. albicans* was the most frequent species (64.6%), followed by *C. tropicalis*, *C. glabrata*, *C. parapsilosis*, and *C. auris*. The antifungal profile demonstrated high sensitivity to voriconazole, caspofungin, and amphotericin B.

Implications. Knowledge of the resistance patterns of predominant *Candida* species in hospitalized patients will strengthen the surveillance of *Candida* resistance.

## MATERIALS AND METHODS

An observational, descriptive, and cross-sectional study was conducted based on the secondary analysis of microbiological and clinical-epidemiological data corresponding to *Candida* isolates obtained from hospitalized patients with a confirmed diagnosis of COVID-19 between November 2020 and April 2022, given that in Peru the first wave of COVID-19 concluded toward the end of 2020 and major waves of cases followed until early 2022, with a high hospital burden and use of antimicrobial treatments.

Identification of the strains was performed using matrix-assisted laser desorption/ionization time-of-flight mass spectrometry (MALDI-TOF MS) using the Bruker Biotyper® system, following the manufacturer's instructions ^15^^,^^16^ and adapting existing protocols for yeast identification. Antifungal susceptibility was evaluated by standardized methods. The unit of analysis was the yeast strain preserved in the mycology collection of the National Reference Laboratory (LRN) of Mycology of the National Institute of Health (INS).

### Population and sample

The strains corresponded to isolates referred to the LRN of Mycology of the INS by various hospitals for precise diagnosis of complex, rare, or severe fungal infections requiring specialized identification and antifungal susceptibility evaluation. The population consisted of all viable strains isolated from various clinical samples (urine, respiratory secretions, blood cultures, among others), and met the following conditions. Inclusion criteria comprised yeast strains of the genus *Candida*, isolated from hospitalized patients with a confirmed diagnosis of COVID-19. Furthermore, only those viable strains preserved in the collection of the National Reference Laboratory of Mycology using the Castellani method were considered. To avoid duplication bias, only one isolate per patient was included. On the other hand, strains that were not viable at the time of analysis, as well as repeated isolates from the same patient, were excluded from the study.

### Data collection

Microbiological and clinical-epidemiological records stored in the LRN of Mycology, corresponding to routine diagnosis, were reviewed. Data were collected from the laboratory's sample reception and processing logbooks, as well as from the epidemiological forms of the received samples, in association with the Netlab 1 system database. The data obtained were recorded in forms designed specifically for the study.

A data cleansing and standardization process was performed, which included the elimination of duplicates, redundant strains, and irrelevant fields; the unification of formats through anonymized forms; the homogenization of dates; and quality control in Excel, discarding incomplete data. Finally, a pilot test was conducted with 50 records to evaluate the clarity of the data collection form, verify the availability of variables in the original records, and standardize extraction criteria before final collection.

### Identification

Two identification methods were used: phenotypic identification by chromogenic agar and mass spectrometry. For phenotypic identification, strains meeting the inclusion criteria were sown on selective and differential medium and incubated at 37 °C for 48 hours, optimal conditions for the formation of colors and distinctive characteristics of each *Candida* species.

For specific and reliable identification, the MALDI-TOF technique was used, considered a standard in clinical microbiology; for this, the manufacturer's instructions ^15^ were followed and existing protocols for yeast identification were adapted. Yeast strains were inoculated into 3 ml of Sabouraud broth and incubated at 37 °C for 24 hours, then a 1.5 ml aliquot of the culture pellet was transferred to Eppendorf tubes. The pellet underwent 2 washes with 1 ml of HPLC-grade water followed by vortex mixing; subsequently, 900 µl of absolute ethanol was added to precipitate proteins, vortexed, and centrifuged at 13,000 rpm for 2 minutes. The supernatant was discarded and the sediment was dried at 37 °C for 10 to 15 minutes to remove ethanol residues. Next, formic acid and acetonitrile were added in equal amounts and a final centrifugation was performed. Finally, 1 µl of the supernatant was dispensed onto the MALDI-TOF plate, and 1 µl of HCCA matrix was added, introducing the plate into the Maldi Biotyper equipment for reading and comparison of the protein spectrum.

A descriptive comparison of the results obtained using chromogenic agar and MALDI-TOF mass spectrometry was performed to identify coincidences and discrepancies in the determination of *Candida* species.

### Susceptibility

Susceptibility determination was performed using two methods: disk diffusion (Kirby-Bauer) and broth dilution. For both procedures, the *Candida krusei* ATCC 6258 strain was used as quality control.

The disk diffusion method was performed according to recommendations of the reference document Clinical and Laboratory Standards Institute (CLSI) M44-A ^17^. From a 24-hour pure culture at 37°C on Sabouraud agar, a suspension adjusted to 0.5 on the McFarland scale was prepared using 0.85% saline solution. Subsequently, this suspension was inoculated onto the surface of Muller Hinton II agar plates, on which specific antifungal susceptibility disks for amphotericin B (20 µg), fluconazole (25 µg), voriconazole (1 µg), and caspofungin (5 µg) were placed. Finally, the plates were incubated for 24 hours at 37 °C. Inhibition diameters were interpreted according to the breakpoints established by CLSI M44, being classified as susceptible (S), susceptible-dose dependent (SDD), intermediate (I), or resistant (R), as appropriate.

The microdilution method was based on the CLSI M27-A3 standard ^18^. Stock solutions were prepared with specific antifungals and dimethyl sulfoxide (DMSO) to achieve the following concentrations: amphotericin B: 0.013 - 16 µg/mL, fluconazole: 0.125 - 64 µg/mL, voriconazole: 0.013 - 16 µg/mL, and caspofungin: 0.015 - 8 µg/mL. These antifungals were selected because they correspond to the most frequent clinical agents in the management of invasive candidiasis and have standardized methodologies and interpretations established by the CLSI for most *Candida* species. Other antifungals were not included due to limitations in the availability of standardized panels and the absence of uniform interpretive criteria for all analyzed species.

Dilutions of each antifungal were placed in plates, leaving the last column free, which was used as a negative control and was dispensed only with DMSO. On all columns, including the positive control, the previously standardized test strain was inoculated. Subsequently, the plates were incubated and growth was evaluated by comparing with the control column, which allowed determining the minimum inhibitory concentration (MIC) of each antifungal against each isolate. Values obtained were interpreted according to clinical breakpoints established by the CLSI, being classified as susceptible (S), susceptible-dose dependent (SDD), intermediate (I), or resistant (R).

### Interpretation of results

For both methods, the interpretation of results was performed according to the provisions of the Clinical and Laboratory Standards Institute (CLSI) M27M44S ^19^, in its recent versions, while for *C. auris*, the tentative breakpoints proposed by the Centers for Disease Control and Prevention (CDC) were used. For the genus *Candida*, the classification is susceptible (S), susceptible-dose dependent (SDD), intermediate (I), and resistant (R) against the evaluated antifungals.

For species that do not have specific clinical breakpoints, interpretation using the broth microdilution method was performed using epidemiological cutoff values (ECV) established in the CLSI M59 document ^20^.

Results were recorded in forms corresponding to each technique, ensuring traceability, reproducibility, and quality control.

### Statistical analysis

The data obtained were analyzed and processed in the Microsoft Excel LTSC Professional Plus 2021 and R Studio version 2023.12.1.402 programs.

### Ethical aspects

The study was based on secondary information obtained from the LRN of Mycology records and surveillance forms. To preserve patient confidentiality and anonymity, all personal data were omitted, ensuring the protection of sensitive information during the strain characterization process. This study corresponds to project OI 011-2023 approved by the Ethics Committee of the National Institute of Health.

## RESULTS

A total of 260 yeast strains of the genus *Candida* isolated from hospitalized patients (average age of 59.5 years) were included. The sex distribution was 62.3% (n = 76) in males and 37.7% (n = 53) in females. Detailed clinical-epidemiological backgrounds by year, origin, sample type, and hospital area are shown in Table 1.

**Table 1:** Characteristics of Candida strains isolated from hospitalized patients with COVID-19.

Characteristic		N=260 (%)
Year		
	2020	20 (7.7)
	2021	151 (58.1)
	2022	89 (34.2)
Origin		
	Amazonas	1 (0.4)
	Ancash	2 (0.8)
	Apurímac	1 (0.4)
	Arequipa	1 (0.4)
	Ayacucho	4 (1.5)
	Callao	4 (1.5)
	Huancavelica	1 (0.4)
	Huánuco	1 (0.4)
	Ica	3 (1.1)
	Junín	5 (1.9)
	La Libertad	20 (7.7)
	Lima	202 (77.7)
	Lima Provincia	10 (3.8)
	Pasco	3 (1.2)
	Piura	1 (0.4)
	San Martin	1 (0.4)
Sample Type		
	Tracheal aspirate	12 (4.6)
	Sputum	4 (1.5)
	Pleural fluid	1 (0.4)
	Urine	135 (51.9)
	Blood	41 (15.8)
	Bronchial secretion	66 (25.4)
	Pharyngeal secretion	1 (0.4)
Hospital Area		
	Emergency	53 (20.4)
	Hospitalization	106 (40.8)
	ICU	101 (38.8)

Of the 260 isolates analyzed by both methods, a high coincidence in species identification was observed. The most frequent species with coincident results were *Candida albicans* (168), *Candida tropicalis* (62), and *Candida glabrata* (17). Likewise, *Candida auris* (3) and *Candida krusei* (2) were coincidently identified. Discrepancies were observed in a small number of isolates. Four samples not determined by chromogenic agar were identified by MALDI-TOF as *Candida parapsilosis*. Additionally, two discordances were recorded in isolates identified as *Candida tropicalis* by the chromogenic method that corresponded to *Candida guilliermondii* and *Candida parapsilosis* by MALDI-TOF. Finally, unique isolates of *Candida kefyr* and *Candida orthopsilosis* that were not determined by chromogenic agar were identified.

The categorical interpretation of antifungal susceptibility using the disk diffusion and microdilution methods is presented in Tables 2 and 3. Generally speaking, both methods showed a similar distribution of susceptibility categories, with a predominance of sensitive isolates for caspofungin and voriconazole, and a lower proportion of sensitivity for fluconazole. Resistance to fluconazole was more frequent compared to the other antifungals in both methods. Differences observed between methods were slight, and the same global trend was maintained in the categorical classification of isolates.

**Table 2:** Antifungal susceptibility by disk diffusion (DD).

Antifungal	Susceptible n (%)	SDD n (%)	Intermediate n (%)	Resistant n (%)	No Interpretation n (%)
Caspofungin	233 (89.6)	—	5 (1.9)	1 (0.4)	21 (8.1)
Fluconazole	198 (76.1)	22 (8.5)	5 (1.9)	27 (10.4)	8 (3.1)
Voriconazole	223 (85.8)	—	7 (2.7)	7 (2.7)	23 (8.8)

SDD: Susceptible-dose dependent

**Table 3:** Antifungal susceptibility by broth microdilution.

Antifungal	Susceptible n (%)	SDD n (%)	Intermediate n (%)	Resistant n (%)	No interpretation n (%)
Caspofungin	245 (94.2)	—	11 (4.2)	2 (0.8)	2 (0.8)
Fluconazole	173 (66.5)	35 (13.5)	—	47 (18.1)	5 (1.9)
Voriconazole	23 (8.9)	—	24 (9.2)	10 (3.8)	23 (8.8)

SDD: Susceptible-dose dependent

The distribution of antifungal resistance in the most representative species of *Candida*, according to hospital area and sample type, is presented in Table 4. In general, resistance was concentrated mainly in isolates from critical areas, particularly the Intensive Care Unit (ICU), where the highest percentages of fluconazole resistance were observed, highlighting *C. auris* (100%), as well as isolated events of caspofungin resistance in *C. krusei* (100%) and *C. tropicalis* (4.2%).

**Table 4:** Distribution of antifungal resistance in *Candida* species according to hospital area and sample type.

Distribution of resistance		Hospital area			Type of sample		
Species	Antifungal	Emergency n (%)	Hospitalization n (%)	ICU n (%)	Urine n (%)	Blood n (%)	Respiratory secretions n (%)
*Candida albicans*	Caspofungin	0 (0)	0 (0)	0 (0)	0 (0)	0 (0)	0 (0)
	Fluconazole	5 (15.2)	12 (16.7)	8 (12.7)	17 (20.5)	4 (17.4)	4 (8)
	Voriconazole	2 (6.1)	5 (6.9)	2 (3.2)	6 (7.2)	2 (8.7)	1 (2)
*Candida auris*	Caspofungin	0 (0)	0 (0)	0 (0)	0 (0)	0 (0)	0 (0)
	Fluconazole	0 (0)	0 (0)	3 (100)	0 (0)	2 (100)	1 (100)
	Voriconazole	0 (0)	0 (0)	0 (0)	0 (0)	0 (0)	0 (0)
*Candida glabrata*	Caspofungin	0 (0)	0 (0)	0 (0)	0 (0)	0 (0)	0 (0)
	Fluconazole	1 (33.3)	4 (50)	1 (16.7)	3 (37.5)	3 (50)	0 (0)
	Voriconazole	0 (0)	0 (0)	0 (0)	0 (0)	0 (0)	0 (0)
*Candida krusei*	Caspofungin	0 (0)	0 (0)	1 (100)	0 (0)	0 (0)	1 (100)
	Fluconazole	0 (0)	0 (0)	0 (0)	0 (0)	0 (0)	0 (0)
	Voriconazole	0 (0)	0 (0)	0 (0)	0 (0)	0 (0)	0 (0)
*Candida parapsilosis*	Caspofungin	0 (0)	0 (0)	0 (0)	0 (0)	0 (0)	0 (0)
	Fluconazole	0 (0)	1 (33.3)	1 (50)	1 (33.3)	1 (100)	0 (0)
	Voriconazole	0 (0)	0 (0)	0 (0)	0 (0)	0 (0)	0 (0)
*Candida tropicalis*	Caspofungin	0 (0)	0 (0)	1 (4.2)	0 (0)	0 (0)	1 (20)
	Fluconazole	4 (25)	6 (27.3)	1 (4.2)	7 (18.4)	4 (50)	0 (0)
	Voriconazole	0 (0)	1 (4.5)	0 (0)	1 (2.6)	0 (0)	0 (0)

Resistance to fluconazole was also documented in hospitalization and emergency, especially in *C. albicans*, *C. glabrata*, and *C. tropicalis*, evidencing the circulation of resistant isolates outside strictly critical settings. Resistance to voriconazole and caspofungin was infrequent in most evaluated areas.

According to the sample type, the highest percentages of resistance were observed in blood isolates (candidemias), particularly for fluconazole in *C. auris* (100%), as well as in *C. glabrata* and *C. tropicalis* (50%). Variable percentages of fluconazole resistance were recorded in urine samples, while resistance was less frequent in respiratory secretions. No resistant isolates were detected in other sterile fluids, although the number of samples in this category was limited.

**Figure 1:**
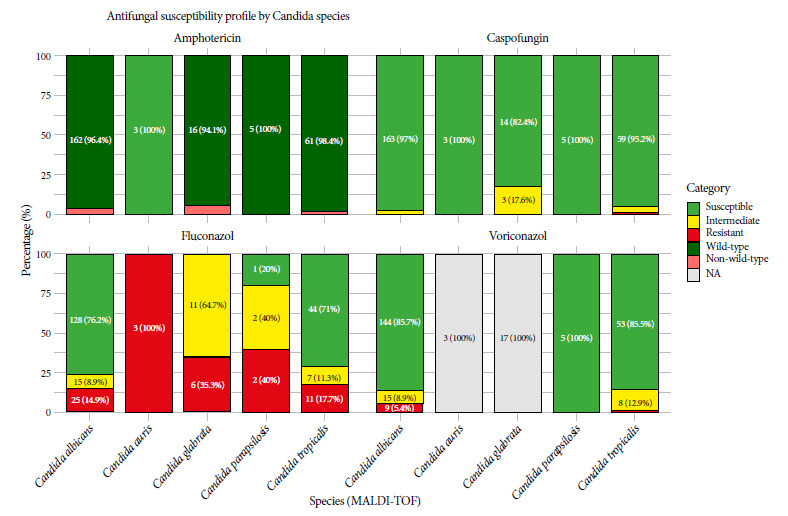
Distribution of antifungal susceptibility categories according to *Candida* species.

The antifungal susceptibility profile of *Candida* isolates was determined from MIC values, interpreted according to CLSI clinical breakpoints for all species, and according to provisional CDC criteria in the case of *C. auris*. The distribution of susceptibility categories is presented in Figure 1.

NA: No interpretation (CLSI)

Interspecies differences were observed in the proportion of sensitive, intermediate, susceptible-dose dependent (SDD), and resistant isolates. For fluconazole, *C. auris* presented a high proportion of resistance, in contrast to *C. albicans*, where the sensitive category predominated. *C. glabrata* showed a relevant proportion of isolates classified as SDD, consistent with its reduced sensitivity profile against azoles.

Regarding caspofungin, most species presented a high proportion of sensitive isolates. However, *C. parapsilosis* evidenced a lower proportion of relative sensitivity compared to other species, in agreement with its lower intrinsic susceptibility to echinocandins.

For voriconazole, the sensitive category predominated in most evaluated species, with low resistance frequency. Similarly, most isolates were classified as sensitive to amphotericin B, without relevant proportions of resistance being observed.

**Figure 2:**
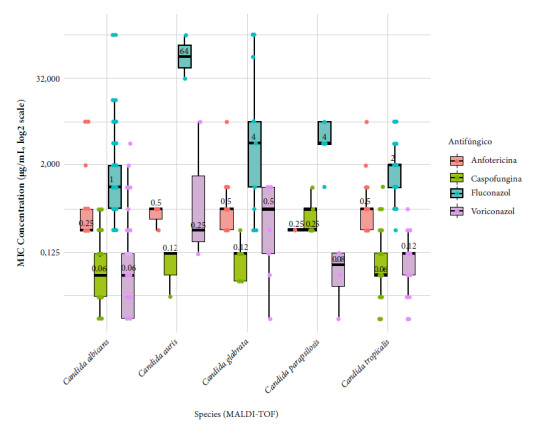
Distribution of minimum inhibitory concentrations (MIC) by microdilution according to Candida species identified by MALDI-TOF.

Taken together, these findings evidence variability in the antifungal susceptibility profile between species, highlighting the high frequency of fluconazole resistance in *C. auris* and the preserved activity of voriconazole and amphotericin B against most isolates. The distribution of minimum inhibitory concentrations (MIC) of the four evaluated antifungals in the five most frequent species of *Candida* is presented in Figure 2.

Interspecies differences in MIC values were evidenced, particularly for fluconazole. *C. auris* showed the highest median for this antifungal (64 µg/mL), notably higher than that observed in *C. albicans* (1 µg/mL), *C. glabrata* (4 µg/mL), and *C. parapsilosis* (4 µg/mL), suggesting lower relative susceptibility in this species.

For caspofungin, medians remained in low ranges (≤0.25 µg/mL) in most species, although *C. parapsilosis* presented slightly higher values compared to the others, a finding consistent with its lower intrinsic sensitivity to echinocandins.

In the case of voriconazole, medians were low and relatively homogeneous between species (≤0.5 µg/mL). Similarly, amphotericin B medians were situated in reduced ranges (0.25-0.5 µg/mL), without relevant increases being observed.

In addition to differences in medians, intraspecies variability in MIC distribution was identified. *C. auris* stands out, presenting a greater dispersion of values against fluconazole, including isolates with high MICs, suggesting heterogeneity in its susceptibility profile. In contrast, for caspofungin and voriconazole, values were concentrated in low ranges, evidencing a more homogeneous distribution among evaluated isolates. The interquartile range was greater in those species-antifungal combinations with higher medians, reflecting greater variability in the antifungal response.

## DISCUSSION

In this study, the phenotypic and resistance profiles of 260 *Candida* strains isolated from hospitalized patients with COVID-19 were characterized. Males predominated (62.3%) and advanced ages (average 59.5 years), in agreement with previous reports associating invasive candidiasis with these groups ^21^^,^^22^. Most isolates came from Lima (77.7%), reflecting hospital centralization and reference bias ^23^. The highest number of cases occurred in 2021, coinciding with COVID-19 hospitalization peaks and intensive use of immunosuppressants ^24^^,^^25^.

Nevertheless, the demographic and clinical profile observed could be influenced by characteristics typical of the population hospitalized for COVID-19 during the pandemic; therefore, these findings should not be interpreted exclusively as an epidemiological pattern inherent to candidiasis, but within the context of the period studied.

The most frequent samples were urine (51.9%), bronchial secretion (25.4%), and blood (15.8%), suggesting the presence of candiduria, possible respiratory colonization, or candidemia reported in hospitalized patients ^26^^,^^27^. The high proportion of urinary isolates could be associated with prolonged use of bladder catheters in hospitalized patients, especially in Intensive Care Units, where candiduria is a frequent finding in the presence of indwelling urinary devices and prolonged hospital stay. Most isolates proceeded from general hospitalization (40.8%) and ICU (38.8%), scenarios where risk factors such as corticosteroid use, immunosuppression, antibiotics, parenteral nutrition, and invasive devices are common ^28^^,^^29^. However, the present study did not specifically evaluate the presence or duration of bladder catheterization, so this possible association must be interpreted with caution.

Identification using chromogenic agar (phenotypic) and MALDI-TOF showed high coincidence, although there were discrepancies in eight strains. MALDI-TOF allowed for more precise identification, highlighting the need to combine conventional methods with high-resolution technologies ^30^. Discrepancies between identification results occur between species such as *Candida haemulonii* and *Candida orthopsilosis*, species that cannot be identified using conventional methods ^31^.

The predominance of *Candida albicans* observed in this study coincides with what has been reported in various Latin American studies on *Candida* infection in hospitalized patients. However, the considerable proportion of non-*albicans* species (35.4%) supports the regional trend toward a progressive increase in these species, a phenomenon associated with intensive use of broad-spectrum antibiotics and selective pressure exerted by antifungals ^32^. In this context, the identification of *Candida auris*, although at low frequency, holds particular epidemiological relevance due to its multidrug resistance capacity, persistence in the hospital environment, and potential to generate nosocomial outbreaks ^12^^,^^33^^,^^34^.

Antifungal resistance profiles, evaluated by disk diffusion (CLSI M44-A) and microdilution (CLSI M27-A3), showed high general coincidence, although the former technique tended to underestimate resistance ^35^. The highest sensitivities were observed against voriconazole and caspofungin, with variable resistances to fluconazole. *C. albicans* was generally sensitive, while *C. krusei* presented resistance or reduced sensitivity, consistent with its known profile ^36^.

*C. auris* isolates, despite being limited, exhibited concerning multidrug resistance profiles, aligned with reports on its difficult management and its capacity to generate intrahospital outbreaks ^37^.

Compared with reports from other Latin American countries, the resistance profile observed in our study shows similarities with systematic reviews that identify variability in azole susceptibility, especially fluconazole, among *Candida* isolates in the Latin American region ^38^. In Peru, recent studies of candidemia have also documented a predominance of non-*albicans* species with high susceptibility to evaluated antifungals ^39^. Conversely, some investigations in Colombia have reported low rates of fluconazole resistance in certain species; however, isolates with resistance mutations have been identified in the country ^40^.

Differences in minimum inhibitory concentrations confirm inter-species variability ^41^^,^^42^. The need for individualized susceptibility testing and continuous surveillance is highlighted to optimize treatments and avoid dissemination of resistant strains ^43^.

Differences in minimum inhibitory concentrations (MIC) between species and within the same species reflect both intrinsic biological variations and differences in efflux pumps, drug targets, and cell permeability; as well as environmental and clinical use pressures that select less susceptible subpopulations. Multicenter studies and surveillance programs have shown temporal changes in the distribution of species causing candidemias and occasional increases in resistance to azoles and echinocandins in non-*albicans* species, confirming that the variability observed is not only laboratory-based but clinical and epidemiological ^44^.

The appearance and expansion of *C. auris* well illustrates this problem: strains with high MICs for fluconazole, although amphotericin B resistance was lower or similar and, in some clades, reduced sensitivity against polyenes and echinocandins, have forced a review of diagnostic, outbreak control, and empirical treatment protocols in hospitals. The rapid emergence and dissemination capacity remind us that continuous surveillance is essential to detect changes in MIC that modify therapeutic recommendations ^45^.

It is important to highlight that MALDI-TOF and the determination of the minimum inhibitory concentration (MIC) fulfill different but complementary roles in the microbiological management of fungal infections. While MALDI-TOF allows for rapid and precise identification at the species level, which is fundamental in the context of emerging species and cryptic complexes, MIC determination provides quantitative information on the antifungal susceptibility profile, indispensable for guiding therapy. In this sense, both methods are not mutually exclusive but rather integrated within modern mycological diagnosis.

The comparison between disk diffusion and microdilution methods showed good coincidence, highlighting the utility of employing both for a more precise evaluation of antifungal resistance.

The comparison between the disk diffusion and microdilution methods showed good agreement, suggesting that both procedures can effectively complement each other for a more reliable evaluation of antifungal resistance. This supports the utility of the combined use of both methods, as joint application allows for the reduction of interpretive discrepancies and improves accuracy in the classification of resistant or susceptible strains. In this context, the combined use of both methods contributes to strengthening the surveillance of antifungal resistance patterns.

This study presents some limitations that should be considered when interpreting the results. First, most of the strains came from the city of Lima, which could induce centralization bias and limit national representativeness. Likewise, because the isolates correspond to strains referred to the LRN of Mycology for diagnostic confirmation or specialized evaluation, it is likely that a reference bias exists with possible overrepresentation of complex cases, suspected resistance, or unusual species. Consequently, results should not be interpreted as representative of all Candida isolates in all hospitals in the country nor allow for estimates of incidence or prevalence.

Secondly, the antifungal resistance evaluation was restricted to drugs with interpretive breakpoints established by the CLSI at the time of analysis, so other frequently used antifungals were not included. Furthermore, for some non-*albicans* species, clinical breakpoints are not yet fully standardized, which may limit the interpretation of MIC values and force the use of reference epidemiological parameters.

Finally, the retrospective design and microbiological nature of the study prevented clinical correlation with therapeutic outcomes, treatment response, or mortality; therefore, findings should be interpreted primarily from a diagnostic and microbiological surveillance perspective.

Nevertheless, despite these limitations, the study provides relevant information on *Candida* identification using MALDI-TOF and its comparison with conventional methods (chromogenic technique); as well as on the antifungal sensitivity profile observed in hospital isolates during the pandemic in Peru.
