# Supplementary material for: Candida species identified by MALDI-TOF and antifungal susceptibility in hospitalized patients with COVID-19 in Peru
Source: Rev Peru Med Exp Salud Publica. 2026 Mar 27;43(1):16–26. doi: 10.17843/rpmesp.2026.431.15685 (PMC13245992; doi:10.17843/rpmesp.2026.431.15685)
Supplement: Translation

# Translation: Especies de Candida identificadas por MALDI-TOF y susceptibilidad antifúngica en pacientes hospitalizados con COVID-19 en Perú

**Authors:** Rolando Paredes-Gago, Sorell Alvarado-Vela, Clelia Céspedes-Román

**Affiliations:** 1 Instituto Nacional de Salud, Centro Nacional de Salud Pública, Lima, Perú.

**Keywords:** Candida, COVID-19, Farmacorresistencia Fúngica Múltiple, Infecciones Oportunistas

## Abstract

**Objetivos.:**

Identificar especies de *Candida* mediante MALDI-TOF y analizar su susceptibilidad antifúngica de aislamientos del género *Candida* en pacientes hospitalizados con COVID-19 entre noviembre de 2020 y abril de 2022.

**Materiales y métodos.:**

Estudio observacional, descriptivo y transversal basado en el análisis secundario de datos microbiológicos y clínico-epidemiológicos de 260 aislamientos de *Candida* provenientes principalmente de orina, secreciones respiratorias y hemocultivos, remitidos al laboratorio de Referencia Nacional de Micología del Instituto Nacional de Salud. La identificación se realizó mediante técnicas fenotípicas y MALDI-TOF, y la susceptibilidad antifúngica se evaluó mediante difusión en disco y microdilución en caldo según criterios del Clinical and Laboratory Standards Institute (CLSI).

**Resultados.:**

*Candida albicans* fue la especie más prevalente (64,6%), seguida de *Candida tropicalis, Candida glabrata*, *Candida parapsilosis* y *Candida auris*. La mayoría de los aislamientos mostró susceptibilidad ha voriconazol y caspofungina, mientras que se observaron porcentajes variables de susceptibilidad a fluconazol, especialmente en *C. glabrata* y *C. auris*. Los valores de la concentración mínima inhibitoria (MIC) evidenciaron variabilidad entre especies y antifúngicos.

**Conclusiones.:**

Las especies no*-albicans* representaron una proporción considerable de los aislamientos analizados. Estos hallazgos describen el patrón de especies y susceptibilidad antifúngica en cepas remitidas al Laboratorio de Referencia Nacional de Micología del Instituto Nacional de Salud durante la pandemia de COVID-19.

## INTRODUCCIÓN

Durante la pandemia por COVID-19 se observó un incremento de infecciones fúngicas en pacientes críticos, especialmente en unidades de cuidados intensivos (UCI) ^1^^,^^2^. Aunque las infecciones por *Candida* se han considerado tradicionalmente oportunistas, en los casos graves la infección parece estar relacionada con factores derivados del manejo clínico que con estados clásicos de inmunosupresión primaria. En particular, la candidiasis invasiva se ha asociado al uso prolongado de antibióticos de amplio espectro y corticosteroides, la ventilación mecánica, la presencia de dispositivos invasivos y la estancia hospitalaria prolongada, condiciones que favorecen la colonización e infección por *Candida* ^(^^3^^-^^5^. Diversos estudios han documentado la presencia de especies como *Candida albicans, Candida glabrata, Candida tropicalis, Candida parapsilosis* y *Candida auris* en pacientes con COVID-19, tanto en casos de colonización como de infección invasiva ^6^.

La candidemia, ha sido reportada en hasta un 8,9% de los pacientes con COVID-19 en UCI, presentando tasas de mortalidad elevadas y la aparición de cepas resistentes a antifúngicos, incluyendo fluconazol y voriconazol ^7^^-^^9^. Estas variaciones en la distribución de especies y en los perfiles de susceptibilidad sugieren cambios en la epidemiología de la candidemia asociada a COVID-19, con implicancias terapéuticas relevantes. En el Perú, la información sobre especies de *Candida* y su perfil de susceptibilidad antifúngica son limitados. Un estudio multicéntrico realizado en 15 hospitales analizó 73 aislamientos de hemocultivos, encontrando que *C. parapsilosis* (53,4%)*, C. albicans* (31,5%) y *C. tropicalis* (6,8%) fueron las especies más prevalentes, y que todos esos aislamientos fueron susceptibles a anfotericina B, fluconazol, voriconazol y anidulafungina ^10^. En la región Lambayeque se reportó que 20% de aislamientos distintos de *C. albicans* mostraron resistencia a fluconazol, y 22% a voriconazol ^11^. Durante la pandemia se notificó por primera vez en el Perú el hallazgo de *C. auris* resistente a fluconazol, en tres pacientes hospitalizados en UCI, lo que sugiere un cambio emergente en la epidemiología local ^(^^12^. En Lima y Callao se documentó una incidencia de 2,04 casos por 1000 admisiones, con predominio de especies no*-albicans* (72%) y baja resistencia a fluconazol (<3%) ^(^^13^.

Aunque el cultivo micológico está ampliamente disponible, el acceso a métodos de identificación a nivel de especie y a pruebas de susceptibilidad antifúngica es limitado fuera de Lima. Además, la disponibilidad de antifúngicos de amplio espectro, particularmente equinocandinas, es restringida en varios establecimientos de salud ^(^^14^, lo que puede dificultar la detección temprana de especies emergentes y de perfiles de resistencia.

El uso masivo e indiscriminado de antimicrobianos durante la pandemia, junto con las condiciones de hacinamiento hospitalario, se considera que favoreció la selección de cepas resistentes y el desplazamiento de especies menos comunes. Asimismo, las alteraciones en la microbiota respiratoria y digestiva inducidas por tratamientos intensivos pudieron propiciar el sobrecrecimiento de levaduras y su posterior invasión tisular.

El presente estudio busca aportar evidencia sobre la distribución de especies de *Candida* y sus perfiles de susceptibilidad antifúngica en un contexto de alta presión hospitalaria, generando información para la vigilancia de infecciones fúngicas asociadas a la atención en salud al proporcionar datos locales que permitan monitorear la tendencia de especies emergentes y patrones de susceptibilidad antifúngica y orientar estrategias de control y prevención de infecciones fúngicas. En este contexto, el estudio tuvo como objetivo identificar las especies del género *Candida*, mediante el método MALDI-TOF, y analizar su susceptibilidad antifúngica en aislamientos obtenidos de pacientes hospitalizados con COVID-19 entre noviembre de 2020 y abril de 2022.

**MENSAJE CLAVE**

Motivación para realizar el estudio. Durante la pandemia por COVID-19, se reportó un aumento de infecciones fúngicas oportunistas; sin embargo, en el Perú no se dispone de estudios descriptivos amplios que documenten las especies de *Candida* y su susceptibilidad antifúngica en el contexto hospitalario.

Principales hallazgos. Los hallazgos demostraron que *C. albicans* fue la especie más frecuente (64,6%), seguida por *C. tropicalis, C. glabrata, C. parapsilosis* y *C. auris.* El perfil de antifúngico demostró una alta sensibilidad ha voriconazol, caspofungina y anfotericina B.

Implicancias en salud pública. Conocer los patrones de resistencias de las especies de *Candida* predominantes en pacientes hospitalizados permitirá fortalecer la vigilancia de la resistencia en *Candida*.

## MATERIALES Y MÉTODOS

Se realizó un estudio observacional, descriptivo y transversal basado en el análisis secundario de datos microbiológicos y clínicos-epidemiológicos correspondientes a asilamientos de *Candida* obtenidos de pacientes hospitalizados con diagnostico confirmado de COVID-19 entre noviembre de 2020 y abril de 2022, dado que en el Perú la primera ola de COVID-19 concluyo hacia finales de 2020 y se sucedieron olas importantes de casos hasta principios de 2022, con elevada carga hospitalaria y uso de tratamientos antimicrobianos.

La identificación de las cepas se realizó mediante espectrometría de masas por desorción/ionización láser asistida por matriz (MALDI-TOF MS) utilizando el sistema Bruker Biotyper®, siguiendo las instrucciones del fabricante ^15^^,^^16^ y adaptando protocolos existentes para la identificación de levaduras. La susceptibilidad antifúngica se evaluó por métodos estandarizados. La unidad de análisis fue la cepa levaduriforme conservada en la micoteca del laboratorio de Referencia Nacional (LRN) de Micología del Instituto Nacional de Salud (INS).

### Población y muestra

Las cepas correspondieron a asilamientos remitidos al LRN de Micología del INS por parte de diversos hospitales para un diagnóstico preciso de infecciones fúngicas complejas, raras o graves que requieren identificación especializada y evaluación de susceptibilidad antifúngica.

La población estuvo conformada por todas las cepas viables aisladas a partir de diversas muestras clínicas (orina, secreciones respiratorias, hemocultivos, entre otras), y cumplieron con las siguientes condiciones.

Los criterios de inclusión comprendieron cepas levaduriformes del género *Candida*, aisladas de pacientes hospitalizados con diagnóstico confirmado de COVID-19. Asimismo, se consideraron únicamente aquellas cepas viables conservadas en la micoteca del Laboratorio de Referencia Nacional de Micología mediante el método de Castellani. Con el fin de evitar sesgos por duplicación, se incluyó un solo aislamiento por paciente. Por otro lado, se excluyeron del estudio aquellas cepas que no se encontraban viables al momento del análisis, así como los aislamientos repetidos provenientes de un mismo paciente.

### Recolección de datos

Se revisaron los registros microbiológicos y clínico-epidemiológicos almacenados en el LRN de Micología, correspondiente a la rutina diagnostica. Los datos fueron recolectados de los cuadernos de registro de recepción y procesamiento de muestras del laboratorio, así como de las fichas epidemiológicas de las muestras recepcionadas, en asociación con la base de datos del sistema Netlab 1. Los datos obtenidos se registraron en fichas diseñadas específicamente para el estudio.

Se realizó un proceso de depuración y estandarización de datos que incluyó la eliminación de duplicados, cepas redundantes y campos irrelevantes; la unificación de formatos mediante fichas anonimizadas; la homogenización de fechas; y el control de calidad en Excel, descartando datos incompletos. Finalmente, se efectuó una prueba piloto con 50 registros con el fin de evaluar la claridad de la ficha de recolección de datos, verificar la disponibilidad de variables en los registros originales y estandarizar los criterios de extracción antes de la recolección definitiva.

### Identificación

Se realizó dos métodos de identificación, la fenotípica por agar cromogénico y por espectrometría de masas. Para la identificación fenotípica, las cepas que cumplían con el criterio de inclusión se sembraron en medio selectivo y diferencial e incubadas a 37 °C por 48 horas, condiciones óptimas para la formación de colores y características distintivas de cada especie de *Candida*.

Para una identificación específica y confiable se utilizó la técnica MALDI-TOF, considerada un estándar en microbiología clínica, para esto se siguieron las instrucciones del fabricante ^15^^)^ y se adaptaron protocolos existentes para la identificación de levaduras. Las cepas levaduriformes fueron inoculadas en 3 ml de caldo Sabouraud y se incubo a 37 °C por 24 horas, luego se alícuota 1,5 ml del pellet del cultivo y se transfirió a tubos Eppendorf, el pellet se sometió a 2 lavados con 1ml de agua grado HPLC seguido de mezcló en vórtex, posteriormente se añadió 900 ul de etanol absoluto para precipitar proteínas, se mezcló en vórtex y se centrifugo a 13,000rpm durante 2 minutos, se descartó el sobrenadante y el sedimento se secó a 37 °C durante 10 a 15 minutos para eliminar restos de etanol. A continuación, se adicionó ácido fórmico y acetonitrilo en igual cantidad y se realizó una última centrifugación. Finalmente, se dispensó 1 ul del sobrenadante sobre la placa MALDI-TOF, y se adicionó 1ul de la matriz HCCA, introduciendo la placa en el equipo Maldi Biotyper para la lectura y comparación del espectro proteico.

Se realizo una comparación descriptiva de los resultados obtenidos mediante agar cromogénico y espectrometría de masas MALDI-TOF, con el fin de identificar coincidencias y discrepancias en la determinación de especies de *Candida*.

### Susceptibilidad

La determinación de la susceptibilidad se realizó mediante dos métodos: difusión en disco (Kirby-Bauer) y dilución en caldo. Para ambos procedimientos se utilizó la cepa *Candida krusei* ATCC 6258 como control de calidad.

El método de disco difusión se realizó según recomendaciones del documento de referencia Clinical and Laboratory Standards Institute (CLSI) M44-A ^(^^17^. A partir de un cultivo puro de 24 horas a 37°C en agar Sabouraud, se preparó una suspensión ajustada a 0,5 en la escala de McFarland usando solución salina al 0,85%. Posteriormente esta suspensión fue inoculada sobre la superficie de placas de agar Muller Hinton II, sobre la cual se colocaron disco para antifungigrama específicos para anfotericina B (20 ug), fluconazol (25 ug), voriconazol (1 ug) y caspofungina (5 ug). Finalmente, las placas se incubaron por 24 horas a 37 °C. Los diámetros de inhibición fueron interpretados conforme a los puntos de corte establecidos por el CLSI M44, clasificándose en susceptible (S), susceptible dependiente de la dosis (SDD), intermedio (I) o resistente (R), según corresponda.

El método de microdilución se basó en la norma CLSI M27-A3 ^18^. Se prepararon soluciones madres con antifúngicos específicos y dimetilsulfóxido (DMSO), para logar las siguientes concentraciones: anfotericina B: 0,013 - 16 µg/mL, fluconazol: 0,125 - 64 µg/mL, voriconazol: 0,013 - 16 µg/mL y caspofungina: 0.015 - 8 µg/mL. Estos antifúngicos fueron seleccionados por corresponder a los agentes de uso clínicos más frecuente en el manejo de candidiasis invasiva y por contar con metodologías estandarizadas e interpretaciones establecidas por el CLSI para la mayoría de las especies de *Candida*. Otros antifúngicos no fueron incluidos debido a limitaciones en la disponibilidad de paneles estandarizados y a la ausencia de criterios interpretativos uniformes para todas las especies analizadas.

Las diluciones de cada antifúngico se dispusieron en placas, dejando libre la última columna, la cual se utilizó como control negativo y fue dispensada únicamente con DMSO. Sobre todas las columnas, incluida la de control positivo se inoculó la cepa problema previamente estandarizada. Posteriormente, las placas fueron incubadas y se evaluó el crecimiento comparando con la columna control, lo que permitió determinar la concentración mínima inhibitoria (MIC) de cada antifúngico frente a cada aislamiento. Los valores obtenidos fueron interpretados conforme a los puntos de corte clínicos establecidos por el CLSI, clasificándose en susceptible (S), susceptible dependiente de la dosis (SDD), intermedio (I) o resistente (R).

### Interpretación de resultados

Para ambos métodos la interpretación de resultados se realizó conforme a lo establecido por el Clinical and Laboratory Standards Institute (CLSI) M27M44S ^19^, en sus versiones recientes, mientras que para *C. auris* se utilizaron los puntos de corte tentativos propuestos por el Centro para el Control y la Prevención de Enfermedades (CDC). Para el género *Candida* la clasificación es susceptible (S), susceptibles dependientes de la dosis (SDD), intermedias (I) y resistentes (R) frente a los antifúngicos evaluados.

Para las especies que no disponen de puntos de corte clínicos específicos, la interpretación mediante el método de microdilución en caldo se realizó utilizando puntos de corte epidemiológicos (ECV) establecidos en el documento CLSI M59 ^20^.

Los resultados se registraron en formularios correspondientes a cada técnica, garantizando la trazabilidad, reproducibilidad y control de calidad.

### Análisis estadísticos

Los datos obtenidos fueron analizados y procesadas en el programa Microsoft Excel LTSC Profesional Plus 2021 y R Studio versión 2023.12.1.402.

### Aspectos éticos

El estudio se basó en información secundaria obtenida de los registros del LRN de Micología y de las fichas de vigilancia. Para preservar la confidencialidad y el anonimato de los pacientes, se omitieron todos los datos personales, garantizando la protección de la información sensible durante el proceso de caracterización de las cepas. Este estudio corresponde al proyecto OI 011-2023 aprobado por el Comité de Ética del Instituto Nacional de Salud.

## RESULTADOS

Se incluyeron 260 cepas levaduriformes del género*Candida* aisladas en pacientes hospitalizados (edad promedio de 59,5 años). La distribución por sexo fue de 62,3% (n = 76) en varones y 37,7% (n = 53) en mujeres. Los antecedentes clínicos-epidemiológicos detallados por año, procedencia, tipo de muestra y área hospitalaria se muestran en la tabla 1.

**Tabla 1:** Característica de las cepas de Candida aisladas de pacientes Hospitalizados con COVID-19.

Característica		N=260 (%)
Año		
	2020	20 (7,7)
	2021	151 (58,1)
	2022	89 (34,2)
Procedencia		
	Amazonas	1 (0,4)
	Ancash	2 (0,8)
	Apurímac	1 (0,4)
	Arequipa	1 (0,4)
	Ayacucho	4 (1,5)
	Callao	4 (1,5)
	Huancavelica	1 (0,4)
	Huánuco	1 (0,4)
	Ica	3 (1,1)
	Junín	5 (1,9)
	La Libertad	20 (7,7)
	Lima	202 (77,7)
	Lima Provincia	10 (3,8)
	Pasco	3 (1,2)
	Piura	1 (0,4)
	San Martin	1 (0,4)
Tipo de muestra		
	Aspirado traqueal	12 (4,6)
	Esputo	4 (1,5)
	Liquido pleural	1 (0,4)
	Orina	135 (51,9)
	Sangre	41 (15,8)
	Secreción bronquial	66 (25,4)
	Secreción faríngea	1 (0,4)
Área hospitalaria		
	Emergencia	53 (20,4)
	Hospitalización	106 (40,8)
	UCI	101 (38,8)

De los 260 aislamientos analizados por ambos métodos, se observó una alta coincidencia en la identificación de especies. Las especies más frecuentes con resultados coincidentes fueron *Candida albicans* (168), *Candida tropicalis* (62) y *Candida glabrata* (17). Asimismo, se identificaron de manera coincidente *Candida auris* (3) y *Candida krusei* (2). Se observaron discrepancias en un número reducido de aislamientos. Cuatro muestras no determinadas por agar cromogénico fueron identificadas por MALDI-TOF como *Candida parapsilosis*. Además, se registraron dos discordancias en aislamientos identificados como *Candida tropicalis* por el método cromogénico que correspondieron a *Candida guilliermondii* y *Candida parapsilosis* por MALDI-TOF. Finalmente, se identificaron aislamientos únicos de *Candida kefyr* y *Candida orthopsilosis* que no fueron determinados por el agar cromogénico.

La interpretación categórica de la susceptibilidad antifúngica mediante los métodos de difusión por disco y microdilución se presenta en las tablas 2 y 3. En términos generales, ambos métodos mostraron una distribución similar de categorías de susceptibilidad, con predominio de aislamientos sensibles para caspofungina y voriconazol, y una menor proporción de sensibilidad para fluconazol. La resistencia a fluconazol fue más frecuente en comparación con los otros antifúngicos en ambos métodos. Las diferencias observadas entre métodos fueron leves y se mantuvo la misma tendencia global en la clasificación categórica de los aislamientos.

**Tabla 2:** Susceptibilidad antifúngica por difusión por disco (DD).

Antifúngico	Sensible	SDD	Intermedio	Resistente	Sin Interpretación
	n (%)	n (%)	n (%)	n (%)	n (%)
Caspofungina	233 (89,6)	-	5 (1,9)	1 (0,4)	21 (8,1)
Fluconazol	198 (76,1)	22 (8,5)	5 (1,9)	27 (10,4)	8 (3,1)
Voriconazol	223 (85,8)	-	7 (2,7)	7 (2,7)	23 (8,8)

SDD: Susceptible dosis dependiente

**Tabla 3:** Susceptibilidad antifúngica por microdilución en caldo.

Antifúngico	Sensible	SDD	Intermedio	Resistente	Sin interpretación
	n (%)	n (%)	n (%)	n (%)	n (%)
Caspofungina	245 (94,2)	-	11 (4,2)	2 (0,8)	2 (0,8)
Fluconazol	173 (66,5)	35 (13,5)	-	47 (18,1)	5 (1,9)
Voriconazol	23 (8,9)	-	24 (9,2)	10 (3,8)	23 (8,8)

SDD: Susceptible dosis dependiente

La distribución de resistencia antifúngica en las especies más representativas de *Candida*, según área hospitalaria y tipo de muestra, se presenta en la tabla 4. En general, la resistencia se concentró principalmente en aislamientos provenientes de áreas críticas, particularmente la Unidad de Cuidados Intensivos (UCI), donde se observaron los mayores porcentajes de resistencia a fluconazol, destacando *C. auris* (100%), así como eventos puntuales de resistencia a caspofungina en *C. krusei* (100%) y *C. tropicalis* (4,2%).

**Tabla 4:** Distribución de resistencia antifúngica en especies de *Candida* según área hospitalaria y tipo de muestra.

Distribución de resistencia		Área hospitalaria			Tipo de muestra		
Especies	Antifúngico	Emergencia	Hospitalización	UCI	Orina	Sangre	Secreciones Respiratorias
		n (%)	n (%)	n (%)	n (%)	n (%)	n (%)
*Candida albicans*	Caspofungina	0 (0)	0 (0)	0 (0)	0 (0)	0 (0)	0 (0)
	Fluconazol	5 (15,2)	12 (16,7)	8 (12,7)	17 (20,5)	4 (17,4)	4 (8)
	Voriconazol	2 (6,1)	5 (6,9)	2 (3,2)	6 (7,2)	2 (8,7)	1 (2)
*Candida auris*	Caspofungina	0 (0)	0 (0)	0 (0)	0 (0)	0 (0)	0 (0)
	Fluconazol	0 (0)	0 (0)	3 (100)	0 (0)	2 (100)	1 (100)
	Voriconazol	0 (0)	0 (0)	0 (0)	0 (0)	0 (0)	0 (0)
*Candida glabrata*	Caspofungina	0 (0)	0 (0)	0 (0)	0 (0)	0 (0)	0 (0)
	Fluconazol	1 (33,3)	4 (50)	1 (16,7)	3 (37,5)	3 (50)	0 (0)
	Voriconazol	0 (0)	0 (0)	0 (0)	0 (0)	0 (0)	0 (0)
*Candida krusei*	Caspofungina	0 (0)	0 (0)	1 (100)	0 (0)	0 (0)	1 (100)
	Fluconazol	0 (0)	0 (0)	0 (0)	0 (0)	0 (0)	0 (0)
	Voriconazol	0 (0)	0 (0)	0 (0)	0 (0)	0 (0)	0 (0)
*Candida parapsilosis*	Caspofungina	0 (0)	0 (0)	0 (0)	0 (0)	0 (0)	0 (0)
	Fluconazol	0 (0)	1 (33,3)	1 (50)	1 (33,3)	1 (100)	0 (0)
	Voriconazol	0 (0)	0 (0)	0 (0)	0 (0)	0 (0)	0 (0)
*Candida tropicalis*	Caspofungina	0 (0)	0 (0)	1 (4,2)	0 (0)	0 (0)	1 (20)
	Fluconazol	4 (25)	6 (27,3)	1 (4,2)	7 (18,4)	4 (50)	0 (0)
	Voriconazol	0 (0)	1 (4,5)	0 (0)	1 (2,6)	0 (0)	0 (0)

En hospitalización y emergencia también se documentó resistencia a fluconazol, especialmente en *C. albicans*, *C. glabrata* y *C. tropicalis*, lo que evidencia la circulación de aislamientos resistentes fuera del ámbito estrictamente crítico. La resistencia a voriconazol y caspofungina fue poco frecuente en la mayoría de las áreas evaluadas.

Según el tipo de muestra, los mayores porcentajes de resistencia se observaron en aislamientos sanguíneos (candidemias), particularmente para fluconazol en *C. auris* (100%), así como en *C. glabrata* y *C. tropicalis* (50%). En muestras de orina se registraron porcentajes variables de resistencia a fluconazol, mientras que en secreciones respiratorias la resistencia fue menos frecuente. No se detectaron aislamientos resistentes en otros líquidos estériles, aunque el número de muestras en esta categoría fue limitado.

El perfil de susceptibilidad antifúngica de los aislamientos de *Candida* se determinó a partir de los valores de MIC, interpretados según los puntos de corte clínicos del CLSI para todas las especies, y de acuerdo con los criterios provisionales del CDC en el caso de *C. auris*. La distribución de las categorías de susceptibilidad se presenta en la figura 1.

**Figura 1:** Distribución de categorías de susceptibilidad antifúngica según especie de *Candida*.

NA: Sin interpretacion (CLSI)

Se observaron diferencias interespecie en la proporción de aislamientos sensibles, intermedios, susceptibles dosis dependiente (SDD) y resistentes. Para fluconazol, *C. auris* presentó una elevada proporción de resistencia, en contraste con *C. albicans*, donde predominó la categoría sensible. *C. glabrata* mostró una proporción relevante de aislamientos clasificados como SDD, consistente con su perfil de sensibilidad reducida frente a azoles.

En relación con caspofungina, la mayoría de las especies presentaron una alta proporción de aislamientos sensibles. Sin embargo, *C. parapsilosis* evidenció una menor proporción de sensibilidad relativa en comparación con otras especies, en concordancia con su menor susceptibilidad intrínseca a las equinocandinas.

Para voriconazol, predominó la categoría sensible en la mayoría de las especies evaluadas, con baja frecuencia de resistencia. De forma similar, la mayor parte de los aislamientos fueron clasificados como sensibles a anfotericina B, sin observarse proporciones relevantes de resistencia.

En conjunto, estos hallazgos evidencian variabilidad en el perfil de susceptibilidad antifúngica entre especies, destacando la elevada frecuencia de resistencia a fluconazol en *C. auris* y la actividad conservada de voriconazol y anfotericina B frente a la mayoría de los aislamientos. La distribución de las concentraciones mínimas inhibitorias (MIC) de los cuatro antifúngicos evaluados en las cinco especies más frecuentes de *Candida* se presenta en la figura 2.

**Figura 2:** Distribución de las concentraciones mínimas inhibitorias (MIC) por microdilución según especie de Candida identificada por MALDI-TOF.

Se evidenciaron diferencias interespecie en los valores de MIC, particularmente para fluconazol. *C. auris* mostró la mediana más elevada para este antifúngico (64 µg/mL), notablemente superior a la observada en *C. albicans* (1 µg/mL), *C. glabrata* (4 µg/mL) *y C. parapsilosis* (4 µg/mL), lo que sugiere una menor susceptibilidad relativa en esta especie.

Para caspofungina, las medianas se mantuvieron en rangos bajos (≤0,25 µg/mL) en la mayoría de las especies, aunque *C. parapsilosis* presentó valores discretamente superiores en comparación con las demás, hallazgo concordante con su menor sensibilidad intrínseca a las equinocandinas.

En el caso de voriconazol, las medianas fueron bajas y relativamente homogéneas entre especies (≤0,5 µg/mL). De manera similar, las medianas de anfotericina B se situaron en rangos reducidos (0,25-0,50 µg/mL), sin observarse incrementos relevantes.

Además de las diferencias en las medianas, se identificó variabilidad intraespecie en la distribución de las MIC. Destaca *C. auris*, que presentó una mayor dispersión de valores frente a fluconazol, incluyendo aislamientos con MIC elevadas, lo que sugiere heterogeneidad en su perfil de susceptibilidad. En contraste, para caspofungina y voriconazol los valores se concentraron en rangos bajos, evidenciando una distribución más homogénea entre los aislamientos evaluados. La amplitud intercuartílica fue mayor en aquellas combinaciones especie-antifúngico con medianas más elevadas, reflejando una mayor variabilidad en la respuesta antifúngica.

## DISCUSIÓN

En este estudio se caracterizaron el perfil fenotípico y de resistencia de 260 cepas de *Candida* aisladas de pacientes hospitalizados con COVID-19. Predominaron los varones (62,3%) y las edades avanzadas (promedio 59,5 años), en concordancia con reporte previos que asocian la candidiasis invasiva a estos grupos ^21^^,^^22^. La mayoría de los aislamientos provinieron de Lima (77,7%), reflejando la centralización hospitalaria y el sesgo de referencia ^23^. El mayor número de casos ocurrió en 2021, coincidiendo con los picos de hospitalización por COVID-19 y el uso intensivo de inmunosupresores ^(^^24^^,^^25^.

No obstante, el perfil demográfico y clínico observado podría estar influenciado por las características propias de la población hospitalizada por COVID-19 durante la pandemia, por lo que estos hallazgos no deben interpretarse exclusivamente como patrón epidemiológico inherente a la candidiasis, sino dentro del contexto del periodo estudiado.

Las muestras más frecuentes fueron orina (51,9%), secreción bronquial (25,4%) y sangre (15,8%), lo que sugiere la presencia de candiduria, posible colonización respiratoria o candidemia reportada en pacientes hospitalizados ^(^^26^^,^^27^. La elevada proporción de aislamientos urinarios podría estar asociada al uso prolongado de sondas vesicales en pacientes hospitalizados, especialmente en Unidades de Cuidados Intensivos, donde la candiduria es un hallazgo frecuente en presencia de dispositivos urinarios permanentes y estancia hospitalaria prolongada. La mayoría de los aislamientos procedieron de hospitalización general (40,8%) y UCI (38,8%), escenarios donde los factores de riesgo como uso de corticoides, inmunosupresión, antibióticos, nutrición parenteral y dispositivos invasivos son comunes ^28^^,^^29^. No obstante, el presente estudio no evaluó de manera específica la presencia ni la duración vesical, por lo que esta posible asociación debe interpretarse con cautela.

La identificación mediante agar cromogénico (fenotípico) y MALDI-TOF mostró alta coincidencia, aunque hubo discrepancias en ocho cepas. MALDI-TOF permitió una identificación más precisa, subrayando la necesidad de combinar métodos convencionales con tecnologías de alta resolución ^30^. Las discrepancias entre los resultados de identificación se dan entre especies como *Candida haemulonii* y *Candida orthopsilosis* especies que no se logran identificar mediante método convencionales ^(^^31^.

El predominio de *Candida albicans* observado en este estudio coincide con lo reportado en diversos estudios latinoamericanos sobre infección por *Candida* en pacientes hospitalizados. No obstante, la proporción considerable de especies no*-albicans* (35,4%) respalda la tendencia regional hacia un incremento progresivo de estas especies, fenómeno asociados al uso intensivo de antibióticos de amplio espectro y a la presión selectiva ejercida por los antifúngicos ^32^. En este contexto , la identificación de *Candida auris*, aunque en baja frecuencia, reviste particular relevancia epidemiológica debido a su capacidad de multirresistencia, persistencia en el ambiente hospitalario y potencial de generar brotes nosocomiales ^(^^12^^,^^33^^,^^34^.

Los perfiles de resistencia antifúngica, evaluados por difusión en disco (CLSI M44-A) y microdilución (CLSI M27-A3), mostraron coincidencia general alta, aunque la primera técnica tendió a subestimar la resistencia ^35^. Las mayores sensibilidades se observaron frente a voriconazol y caspofungina, con resistencias variables a fluconazol. *C. albicans* fue generalmente sensible, mientras que y *C. krusei* presentaron resistencia o sensibilidad reducida, coherente con su perfil conocido ^36^.

Los aislamientos de *C. auris* a pesar de ser limitados exhibieron perfiles preocupantes de multirresistencia, alineados con reportes sobre su difícil manejo y su capacidad de generar brotes intrahospitalarios ^37^.

En comparación con reportes de otros países latinoamericanos, el perfil de resistencia observado en nuestro estudio muestra similitudes con revisiones sistemáticas que identifican variabilidad en susceptibilidad a azoles, especialmente fluconazol, entre aislamientos de *Candida* en la región latinoamericana ^38^. En el Perú, estudios recientes de candidemia también han documentado predominio de especies no-*albicans* con alta susceptibilidad a los antifúngicos evaluados ^(^^39^. Por el contrario, algunas investigaciones en Colombia han reportado tasas bajas de resistencia a fluconazol en ciertas especies, sin embargo, se han identificado aislamientos con mutaciones de resistencia en el país ^40^.

Las diferencias en las concentraciones mínimas inhibitorias confirman la variabilidad inter-especie ^41^^,^^42^. Se resalta la necesidad de pruebas individualizadas de susceptibilidad y vigilancia continua para optimizar tratamientos y evitar la diseminación de cepas resistentes ^43^.

Las diferencias en las concentraciones mínimas inhibitorias (MIC) entre especies y dentro de la misma especie reflejan tanto variaciones biológicas intrínsecas como diferencias en bombas de eflujo, dianas farmacológicas y permeabilidad celular; así como presiones ambientales y de uso clínico que seleccionan subpoblaciones menos susceptibles. Estudios multicéntricos y programas de vigilancia han mostrado cambios temporales en la distribución de especies causantes de candidemias y aumentos puntuales de resistencia a azoles y equinocandinas en especies no-*albicans*, lo que confirma que la variabilidad observada no es sólo de laboratorio sino clínica y epidemiológica ^44^.

La aparición y expansión de *C. auris* ilustra bien este problema: cepas con elevadas MIC para fluconazol, aunque la resistencia a la anfotericina B fue menor o similar y, en algunos clados, reducción de sensibilidad frente a polienos y equinocandinas, han obligado a revisar protocolos de diagnóstico, control de brotes y tratamientos empíricos en hospitales. La rápida emergencia y la capacidad de diseminación nos recuerdan que la vigilancia continua es imprescindible para detectar cambios en MIC que modifiquen recomendaciones terapéuticas ^45^.

Es importante destacar que el MALDI-TOF y la determinación de la concentración mínima inhibitoria (MIC) cumplen roles distintos pero complementarios en el manejo microbiológico de las infecciones fúngicas. Mientras que el MALDI-TOF permite una identificación rápida y precisa a nivel de especie, lo cual es fundamental en el contexto de especies emergentes y complejos crípticos, la determinación del MIC proporciona información cuantitativa sobre el perfil de susceptibilidad antifúngica, indispensable para orientar la terapia. En este sentido, ambos métodos no son excluyentes sino integrados dentro del diagnóstico micológico moderno.

La comparación entre los métodos de disco difusión y microdilución mostró buena coincidencia, lo que sugiere que ambos procedimientos puedan complementarse eficazmente para una evaluación más confiable de la resistencia antifúngica. Esto respalda la utilidad del uso combinado de ambos métodos ya que la aplicación conjunta permite reducir discrepancias interpretativas y mejorar la exactitud en la clasificación de cepas resistentes o susceptibles. En este contexto, el uso combinado de ambos métodos contribuye a fortalecer la vigilancia de los patrones de resistencia antifúngica.

Este presente estudio presenta algunas limitaciones que deben considerarse al interpretar los resultados. En primer lugar, la mayoría de las cepas procedieron de la ciudad de Lima, lo que podría inducir un sesgo de centralización y limitar la representatividad nacional. Asimismo, debido a que los aislamientos corresponden a cepas remitidas al LRN de Micología para confirmación de diagnostica o evaluación especializada, es probable que exista un sesgo de referencia con posible sobrerrepresentación de casos complejos, sospecha de resistencia o especies inusuales. En consecuencia, los resultados no deben interpretarse como representativos de la totalidad de aislamientos de *Candida* en todos los hospitales del país ni permitir estimar incidencia o prevalencia.

En segundo lugar, la evaluación de resistencia antifúngica se restringió a los fármacos con puntos de corte interpretativos establecidos por el CLSI al momento del análisis, por lo que no se incluyeron otros antifúngicos de uso frecuente. Además, para algunas especies no-*albicans*, los puntos de corte clínicos aún no se encuentran completamente estandarizados, lo que puede limitar la interpretación de los valores de MIC y obligar al uso de parámetros epidemiológicos de referencia.

Finalmente, el diseño retrospectivo y el carácter microbiológico del estudio impidieron realizar correlación clínica con desenlaces terapéuticos, respuesta al tratamiento o mortalidad, por lo que los hallazgos deben interpretarse principalmente desde la perspectiva diagnóstica y de vigilancia microbiológica.

No obstante, pese a estas limitaciones, el estudio proporciona información relevante sobre identificación de *Candida* mediante MALDI-TOF y su comparación con métodos convencionales (técnica cromogénica); así como sobre el perfil de sensibilidad antifúngica observado en aislamientos hospitalarios durante la pandemia en el Perú.
